# Content Validity of the CHANT’s French-Language Translation and Cultural Adaptation: A Modified E-Delphi Study

**DOI:** 10.3390/nursrep16070244

**Published:** 2026-07-15

**Authors:** Omar Portela dos Santos, Fanny de Hepcée, Paulo Jorge Pereira Alves, Henk Verloo

**Affiliations:** 1 Department of Nursing Sciences, School of Health Sciences, HES-SO Valais/Wallis, University of Applied Sciences and Arts Western Switzerland, Chemin de l’Agasse 5, CH-1950 Sion, Switzerland; 2 Faculty of Health Sciences and Nursing, Catholic University of Portugal, Rua de Diogo Botelho 1327, 4169-005 Porto, Portugal; pjalves@ucp.pt; 3 Institute of Higher Education and Research in Healthcare (IUFRS), Faculty of Biology and Medicine, University of Lausanne, 1010 Lausanne, Switzerland; fanny.dehepcee@unil.ch (F.d.H.); henk.verloo@hevs.ch (H.V.); 4 Centre for Interdisciplinary Research in Health (CIIS–Wounds Research Lab), Catholic University of Portugal, Rua de Diogo Botelho 1327, 4169-005 Porto, Portugal; 5 Service of Old Age Psychiatry, Department of Psychiatry, Lausanne University Hospital, Route de Cery 60, CH-1011 Lausanne, Switzerland

**Keywords:** eco-literacy, eco-responsibility, eco-centricity, nursing sciences, climate change, psychometric validity, content validity index, e-Delphi survey, CHANT

## Abstract

**Background/Objectives:** Climate change is a major global health challenge with direct implications for public health. As frontline health professionals and agents of change, nurses must develop competencies to address climate-related health issues and implement sustainable practices. This study aimed to translate and culturally adapt the Climate, Health, and Nursing Tool (CHANT) into French and to assess its content validity using item-level and scale-level content validity indices (I-CVI and S-CVI, respectively). The CHANT evaluates nurses’ awareness, motivations, concerns and self-reported behaviours related to climate change. A secondary objective was to examine potential associations between experts’ sociodemographic and professional characteristics and their CVI ratings. **Methods**: A descriptive international study using a three-round modified e-Delphi approach was conducted between January and June 2025 in French-speaking regions of Switzerland, France and Belgium. A multidisciplinary panel of experts in nursing, planetary health and environmental sciences evaluated the relevance, clarity and comprehensiveness of each item and response option, enabling iterative refinement. **Results**: The original 12-item CHANT was evaluated by 17 experts in Round 1. Following expert recommendations, one additional item was incorporated, resulting in a 13-item version, which was subsequently evaluated by 15 experts in Round 2 and 25 experts in Round 3. Across the three Delphi rounds, 57 completed expert evaluations contributed to the iterative refinement of the instrument. Round 1 I-CVI ranged from 0.79 to 1.0, and S-CVI reached 0.935, with full consensus. Round 2 I-CVI ranged from 0.71 to 1.0, and S-CVI was 0.91 (92% consensus), with one item not meeting the predefined threshold. Round 3 I-CVI ranged from 0.82 to 1.0, and S-CVI returned to 0.935, confirming consensus. A final linguistic, semantic and cultural review conducted by the research team ensured the conceptual consistency and cultural appropriateness of the translated instrument. **Conclusions**: The French-language version of the CHANT demonstrated satisfactory content validity and provides a culturally adapted instrument to assess climate-related competencies and eco-literacy in nursing education and practice. Further psychometric evaluation is warranted.

## 1. Introduction

Climate change necessitates immediate, coordinated action across every sector of society. Building a climate-competent health workforce is essential to mitigating the health impacts of climate change. Nurses, who represent nearly 60% of the global health workforce and are among the most trusted health professionals, occupy a strategic position from which to influence public health and contribute to the United Nations’ Sustainable Development Goals [1]. The International Council of Nurses Code of Ethics for Nurses emphasises their responsibility to acquire and disseminate knowledge on the health and ecological consequences of climate change, while integrating environmentally responsible clinical and organisational practices [2]. To do so, nurses require new competencies to become eco-literate, eco-responsible and eco-centric practitioners. Eco-literacy encompasses the knowledge, attitudes and behaviours needed to sustain ecosystems and health, pushing them from nominal towards functional and operational levels of engagement [3,4]. Eco-responsibility refers to integrating environmental, social and economic sustainability into professional practice through accountability, proactive action and resilience [5]. Beyond this, adopting an eco-centric worldview enables nurses to transcend anthropocentric paradigms, recognise the interdependence of living systems and embrace planetary stewardship as an ethical imperative [5,6,7].

As frontline healthcare workers and social actors, nurses can play a crucial role in addressing climate-related health risks through global nursing. Global nursing is defined as the application of evidence-based nursing practices that advance planetary health and health equity across populations. It integrates the social determinants of health and encompasses care at both the individual and population levels through clinical practice, research, education, leadership, advocacy and policy engagement [8]. Although assessing nurses’ environmental awareness is vital for advancing healthcare sustainability, evidence shows that they exhibit only moderate levels of knowledge, concern and climate-related behaviours [6,9,10]. Studies have also revealed a limited understanding of healthcare’s own contribution to climate change [11,12,13]. The CHANT survey confirmed moderate awareness (M = 2.97/4) but only low levels of climate-related action in clinical practice (M = 1.81) despite high levels of concern (M = 3.43) [14,15].

Previous studies have demonstrated that the CHANT is a valuable instrument for assessing nurses’ environmental awareness regarding climate change [15,16]. Beyond identifying current levels of climate-related competencies, the CHANT has also shown its utility in highlighting educational needs, informing sustainability initiatives, supporting curriculum development and evaluating interventions designed to strengthen environmental and planetary health competencies among healthcare professionals [15,16]. The CHANT, developed by Schenk et al. in 2019 [15,16], assesses nurses’ awareness, motivations, concerns and self-reported behaviours related to climate change in both their professional and personal contexts. Although the CHANT was developed primarily as a measurement instrument, its conceptual framework is grounded in the Integrated Change Model. This model states that behavioural change results from interactions between awareness factors, motivational determinants and behavioural processes. More specifically, attitudes, self-efficacy and social influences are considered key determinants shaping motivation and behavioural intentions. Within this framework, environmentally sustainable behaviours require more than knowledge alone and involve multidimensional competencies that integrate cognitive, emotional and behavioural components. The CHANT operationalises these dimensions. Consequently, the instrument reflects a progression from healthcare professionals’ climate-related knowledge and perceptions towards their engagement in environmentally sustainable practices [15,16]. An exploratory factor analysis supported a five-factor structure, with a good model fit (CFI = 0.95; RMSEA = 0.04; SRMR = 0.09) and acceptable-to-high internal consistency across subscales (Cronbach’s alpha ranged from 0.69 to 0.91). The CHANT questionnaire’s items use a five-point Likert scale [15,16]. Awareness is evaluated using five items that assess respondents’ familiarity with the health impacts of climate change, with response options ranging from 1 (‘I’ve never heard of it’) to 5 (‘I’ve definitely heard of it’). Concern is measured using five items that gauge respondents’ degree of worry about climate change, rated from 1 (‘Not at all’) to 5 (‘Extremely’). Motivation to act is assessed using three items, with responses ranging from 1 (‘Never’) to 5 (‘Always’). Ecological behaviour is evaluated using two sets of items—five items for behaviours at home and four for behaviours at work—both rated on a scale from 1 (‘Never’) to 5 (‘Always’).

Despite its demonstrated utility and promising psychometric properties, the CHANT was unavailable in French. The absence of a culturally adapted French-language version constituted a significant methodological and practical gap. This limitation restricted the assessment of eco-literacy and climate-related competencies among French-speaking healthcare professionals and hindered both international comparisons and the implementation of educational and clinical initiatives. Assessing content validity is a key step in the cultural adaptation of measurement tools for nursing research. Using both an item-level content validity index (I-CVI) and a scale-level content validity index (S-CVI) provides a rigorous method of ensuring item relevance and clarity. This is particularly important for instruments like the CHANT, which target emerging concepts such as nurses’ eco-literacy and require strong psychometric foundations to support their use in research, education and clinical practice. Therefore, the present study aimed to translate and culturally adapt the CHANT into French and to establish expert panel consensus on both its I-CVI and its S-CVI. A secondary objective was to examine potential associations between experts’ sociodemographic and professional characteristics and their CVI assessments.

## 2. Materials and Methods

### 2.1. Design

To address our research objectives, we used a modified, three-round, multicentre e-Delphi method guided by the Conducting and REporting DElphi Studies (CREDES) framework developed by Jünger et al. [17] This framework guarantees the rigour and transparency of this type of consensus-seeking approach [18]. The e-Delphi method [19] was selected for its methodological rigour, flexibility and capacity to facilitate anonymous, asynchronous expert input through an iterative process. Unlike classic Delphi studies, which aim to assess changes in the opinions of the same experts across successive rounds, modified Delphi approaches, developed for questionnaire translation, cross-cultural adaptation and content validation, focus primarily on refining instrument content through iterative expert feedback. Consequently, retaining identical participants throughout the process is not an essential methodological requirement. Instead, the emphasis is on ensuring that experts across each round possess comparable levels of expertise and represent the relevant professional domains. In the present study, all the participants met our predefined eligibility criteria, thereby ensuring consistency in their multidisciplinary expertise while allowing flexibility in panel composition. This approach is considered methodologically appropriate for instrument development and content validation studies, where the primary objective is to optimise item relevance, clarity and comprehensiveness rather than to evaluate longitudinal changes in individual opinions. Accordingly, panel variability should be interpreted as an inherent characteristic of a modified Delphi design rather than as a methodological deviation [18,19,20]. Finally, after completing the three rounds, we conducted a final linguistic, semantic and cultural review involving members of the research team. This review aimed to ensure conceptual consistency, semantic equivalence and cultural appropriateness of the translated questionnaire after integration of expert feedback [21,22,23].

The original English-language CHANT comprised 12 items. During the modified e-Delphi process, experts were invited to propose additional content if important dimensions appeared to be missing. Following Round 1, one new item addressing climate-related concerns was added based on recurring expert recommendations. Consequently, Rounds 2 and 3 evaluated a 13-item version of the instrument.

### 2.2. Translation and Linguistic Adaptation Procedure

The translation and cultural adaptation process followed internationally recognised recommendations for the cross-cultural adaptation of self-reporting instruments [21,22,23,24,25]. First, the original English-language CHANT questionnaire was translated into French by two fluent bilingual translators familiar with healthcare terminology. Their independently translated versions were then compared and combined through discussions by the research team (OPDS and HV) to produce a single preliminary French-language version. Discrepancies in wording, terminology and conceptual interpretation were reviewed to ensure semantic and conceptual equivalence rather than a literal translation. A backward translation into English was then performed by an independent bilingual translator with no prior involvement in the study and blinded to the original questionnaire. The back-translated version was compared with the original CHANT instrument to identify potential inconsistencies or conceptual deviations. Any discrepancies were resolved through a consensus-based discussion involving a third member of the research team (FDH). Finally, some terminological refinements that emerged from our expert consensus were incorporated into the adapted questionnaire, all the while maintaining consistency with the terminology of the original CHANT and the broader scientific literature.

### 2.3. Data Collection

In contrast to traditional Delphi rounds, a real-time e-Delphi approach enables continuous expert interactions within defined timeframes using specialised software [23,24,25] (see Table 1). Our modified online format was implemented using SurveyMonkey software, which significantly streamlined administration.

### 2.4. Sample

Selecting the expert panel is a critical component of any Delphi study. As Sablatzky [23] highlighted, while representativeness is not required, participants must be engaged, knowledgeable and well-informed, as their expertise and potential biases can substantially influence outcomes. Given the absence of any universally accepted criteria for defining expertise in Delphi studies, expert selection was based on predefined eligibility criteria to ensure methodological credibility, confirmability and consistency. Participants were recruited according to: (1) their knowledge and/or professional experience in healthcare, sustainability, climate change or planetary health; (2) their residency in French-speaking Switzerland, France or Belgium; and (3) their professional experience in the healthcare sector. This approach aimed to maximise the relevance and diversity of perspectives while ensuring consistency across study rounds [18,25]. French-speaking Switzerland, France and Belgium were selected because, despite sharing a common language, they differ in healthcare organisation, educational structures, professional practices and the sociocultural perspectives that may influence the interpretation of climate-related concepts. Including experts from these contexts aimed to improve the linguistic equivalence, cultural sensitivity and transferability of the adapted French-language version of the CHANT.

### 2.5. Statistical Analysis

In e-Delphi studies, consensus is typically defined by the proportion of experts endorsing an item [26]. A priori thresholds commonly range from 51% to 100%; a 75% agreement level is frequently adopted [25,26]. Data were analysed using SPSS statistical software, v29.0 (IBM Corp., Chicago, IL, USA). Descriptive statistics summarised the experts’ sociodemographic and professional characteristics. Continuous variables are presented as medians and interquartile ranges (IQR), whereas categorical variables are reported as frequencies and percentages. Content validity was assessed using the I-CVI and the S-CVI based on the average method (S-CVI/Ave). Items with an I-CVI ≥ 0.78 were considered acceptable, whereas items below this threshold were revised according to expert feedback. An S-CVI/Ave ≥ 0.90 indicated acceptable scale content validity. Although several methods exist for assessing content validity, including Lawshe’s Content Validity Ratio, modified kappa coefficients, Aiken’s V or Rasch-based indices, we selected the I-CVI/S-CVI approach within a modified e-Delphi framework because it combines quantitative agreement indices with iterative qualitative feedback, making it particularly suitable for questionnaire translation, cross-cultural adaptation and content validation [26].

Qualitative comments were analysed using an inductive open-coding approach. Two authors (OPDS and FDH) independently coded every comment according to the type of modification proposed (e.g., wording clarification, conceptual clarification, terminology refinement, response option modification, additional concepts and cultural adaptation). Codes were then grouped into broader categories through discussion, and disagreements were resolved by consensus with a third author (HV). Item revisions were retained when supported by expert feedback and judged to improve semantic equivalence, conceptual consistency, cultural appropriateness or content relevance. Formal inter-coder agreement statistics were not calculated because the qualitative analysis was intended to support questionnaire refinement rather than constitute a standalone qualitative study. As the data were not normally distributed (Shapiro–Wilk test) and sample sizes were small, we performed non-parametric analyses. We used Mann–Whitney U tests to examine differences by sex, Kruskal–Wallis tests for educational level, profession and country of residence, and Spearman’s rank correlation coefficients for associations between age, questionnaire completion time and I-CVI ratings. Statistical significance was set at α = 0.05. Because these inferential analyses were predefined as exploratory secondary analyses, no correction for multiple comparisons was applied; consequently, the findings should be interpreted cautiously as hypothesis-generating rather than confirmatory.

## 3. Results

### 3.1. Sample Characteristics

Of the 233 individuals invited to participate in the study, 117 (50.2%) began one round of the questionnaire, and a total of 57 completed expert evaluations were obtained across the three Delphi rounds (17 in Round 1, 15 in Round 2 and 25 in Round 3). Because panel membership varied across rounds, these evaluations did not necessarily correspond to 57 unique experts. Questionnaire completion rates among the expert participants who began one were 80.6% (17/21) in Round 1, 88.2% (15/17) in Round 2 and 100% (25/25) in Round 3. Because different experts participated across different e-Delphi rounds, participants’ characteristics were analysed separately for each one. Although panel membership varied, all experts fulfilled the same predefined eligibility criteria, ensuring comparable expertise while maintaining multidisciplinary representation throughout the study. Most of the experts resided in Switzerland (55.0%), followed by France (43.3%) and Belgium (1.7%). The overall median expert age was 42 years old (IQR = 23), ranging from 20 to 74. Most participants were nurses (31.6%) or physicians (21.1%), with contributions from midwives, environmental specialists, allied health professionals, educators and university students. The variability observed in the experts’ characteristics was intentional and reflected the multidisciplinary nature of the study topic. Given that climate change and sustainability involve clinical, educational, environmental and public health dimensions, experts from different professional and educational backgrounds were included to maximise the diversity of perspectives while maintaining our predefined eligibility criteria (see Table 2).

In Round 1 (n = 17), 13 participants were based in Switzerland (76.5%), with 2 each in France and Belgium. The sample was predominantly female (58.8%), with a median age of 40 years old (range = 26–73 years; IQR = 17.5), indicating that the panel had broad professional experience. The median questionnaire completion time was 21.9 min (IQR = 21.4), excluding two extreme outliers (at >1440 min and >10,080 min) attributed to asynchronous completion. Participants held a diverse range of healthcare and sustainability roles categorised into five groups for analysis: nurses, physicians, allied health professionals, health educators/clinical instructors and sustainability/environmental specialists. Nurses made up the largest group (29.4%), followed by sustainability/environmental specialists and allied health professionals (23.5% each), physicians (17.6%) and educators (5.9%) (Table 2).

Round 2 participants also primarily resided in Switzerland (86.7%), with a smaller number in France (13.3%) and none in Belgium. Most were female (73.3%), and most were nurses (33.3%), followed by physicians and sustainability/environmental specialists (20.0%), midwives (13.3%), and others (6.7%). Regarding their level of education, most held a bachelor’s degree (60.0%), followed by those with a PhD (20.0%) or a master’s degree (20.0%). No participants reported having a continuing education certificate. The median age was 43 years old (range = 22–74; IQR = 23). The median questionnaire completion time was 25.47 min (IQR = 34.35) (Table 2).

Finally, in Round 3 (n = 25), again most participants were from Switzerland (56%) and France (40%), with one from Belgium (4%). Median age was 42 years old (range = 20–67; IQR = 23). The median questionnaire completion time was 10.6 min (IQR = 21.2 min). One participant took over a week to complete the questionnaire, but was considered an outlier and excluded, as in Round 1. Professions included nurses (28%), physicians (28%), educators (12%), sustainability specialists (12%), allied health professionals (4%) and one nursing student (4%). The ‘others’ category (12%) comprised a manager, an economist and a healthcare director. Among the nurses, two identified themselves as eco-nurses, healthcare professionals who have undergone a relevant continuing education course to understand and address the connections between environmental factors and human health. Their roles include raising awareness of environmental health risks (e.g., air pollution, endocrine disruptors), educating patients and peers on sustainable practices, promoting eco-responsible approaches in clinical settings and advocating for health policies that incorporate ecological concerns. Although the title of eco-nurse is not yet officially recognised in most French-speaking countries, specialised continuing education programmes are available in some regions. France’s Institute for Training in Environmental Health (known by the acronym IFSEN) offers a 189 h hybrid training course on Environmental Health: Theory and Practice, which covers key topics such as toxicology, ecology, sustainability and integrative health [27]. Round 3 comprised 36% of respondents with a master’s degree, 28.0% with a bachelor’s degree, 24.0% with a PhD, and 12.0% had completed some specialised continuing education (Table 2).

Overall, participant characteristics varied modestly across the three Delphi rounds while maintaining comparable multidisciplinary expertise. Nurses and physicians remained the predominant professional groups, and most experts held postgraduate qualifications, ensuring consistency with the predefined eligibility criteria despite variations in panel composition.

### 3.2. Research Question Results

Following Round 1, recurring expert feedback identified the absence of an item specifically assessing concern about climate change. Consequently, a new item (the 13th) was added within the *concern* domain to capture this dimension. This additional item became Item 2 in the subsequent Delphi rounds.

**Figure 1:**
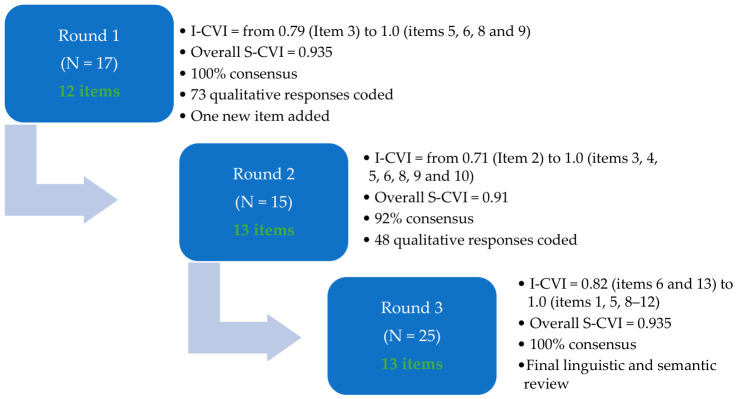
Reactive e-Delphi process rounds and results.

In Round 1 (Figure 1), I-CVI ratings ranged from 0.79 (Item 3) to 1.0 (items 5, 6, 8, 9), indicating strong general agreement among the experts regarding item relevance. At 0.935, the average S-CVI (S-CVI/Ave) was high. In Round 2 (Figure 1), I-CVI ratings ranged from 0.71 (Item 2, just added) to 1.0 (items 3–6, 8, 9 and 10), with Item 3 showing the most substantial improvement between rounds 1 and 2 (0.79 vs. 1.0). In Round 3 (Figure 1), I-CVI ratings ranged from 0.82 (items 6 and 13) to 1.0 (items 1, 5 and 8–12). The S-CVI/Ave returned to 0.935.

Rounds 1 and 3 (Figure 1 and Table 3) both attained levels of 100% consensus on key decisions. Consensus was not reached in Round 2 due to the I-CVI for Item 2 (0.71). Although I-CVI ratings fluctuated for specific items—such as the notable increases for items 11 (0.87 vs. 0.90 vs. 1.0) and 12 (0.86 vs. 0.94 vs. 1.0), and the gradual decline for Item 7 (0.92 vs. 0.90 vs. 0.88)—overall content validity remained consistently strong. Items 5, 8 and 9 maintained an I-CVI of 1.0 in rounds 1 and 2 (Figure 1).

The experts assessed the items’ relevance (Table 3) on a Likert scale ranging from 1 to 4: 1 (*irrelevant*), 2 (*slightly relevant*), 3 (*relevant*) and 4 (*highly relevant*). In Round 1, Item 5 had the highest mean rating of 3.79 and the most favourable evaluation in the set. Conversely, items 3 and 12 had the lowest mean ratings, both at 3.07. The stringency of each expert was further explored by examining the frequency with which low relevance ratings (1 or 2) were assigned. This analysis highlighted differences in rating stringency across experts. Expert 1 was strict, assigning a rating of *irrelevant* to Item 6. Experts 3 and 9 exhibited greater overall stringency, each assigning ratings of *slightly relevant* to three distinct items. Expert 13 was also moderately severe, rating two items as *slightly relevant*. This analysis highlighted variations in experts’ stringency during the relevance evaluation process. In Round 2, items 3, 4, 5, 9 and 11 had the highest mean rating (M = 3.67), whereas Item 1 had a mean rating of 3.11. The newly added Item 2 had a mean relevance rating of 3.33. In Round 3, Item 10 had the highest mean rating (M = 3.78) and Item 2 had the lowest (M = 3.00). The most severe evaluators were Expert 3 (who assigned one rating of *irrelevant* and one of *slightly relevant*), Expert 2 (who assigned three ratings of *slightly relevant*) and Expert 15 (who assigned two ratings of *slightly relevant*).

The experts rated the comprehension of each item (Table 4) on a three-point scale: 1 (*comprehensible*), 2 (*somewhat comprehensible*) and 3 (*not comprehensible*). Item 1 was unanimously rated as *comprehensible*. However, some variability was noted for other items: one expert rated items 6, 7, 8 and 12 as *somewhat comprehensible*, and two experts assigned this rating to Item 11. Three experts also rated items 2, 3, 9 and 10 as *somewhat comprehensible*, while Item 4 received that rating from four experts. Notably, a single expert rated items 5, 7, 8 and 9 as *not comprehensible* (rating 3) and suggested corresponding reformulations. In Round 2, all the experts rated items 1, 3, 4, 6, 8, 9, 10 and 13 as *comprehensible*. For the remaining items, a limited number of experts still reported minor comprehension concerns. One expert rated Items 2, 5, 11, and 12 as *somewhat comprehensible*. Item 7 was rated *not comprehensible* by two experts. In Round 3, Item 10 was unanimously rated as *comprehensible*. One expert rated Items 3, 7, 9, 11, 12 and 13 as *somewhat comprehensible*, whereas Items 5, 6 and 8 were rated as *somewhat comprehensible* by three experts. Item 4 received the highest number of *somewhat comprehensible* ratings, with six experts assigning this rating. No item was rated as *not comprehensible*.

During each Delphi round, our experts were invited to provide qualitative comments on the items and suggest revisions to improve the translated questionnaire. A total of 73 comments were coded in Round 1 and 48 in Round 2. Of these, 78 were language reformulations and 43 added a term, definition, concept or another element across the two rounds, supporting the instrument’s iterative refinement. Comments were coded into six predefined categories: wording clarification, conceptual clarification, terminology refinement, addition of concepts, modification of response options and cultural adaptation. Proposed modifications were incorporated into subsequent questionnaire versions and re-evaluated in the following Delphi round (Table 5).

In Round 1, Item 1 received the most suggestions, primarily aimed at improving comprehensibility through the inclusion of concepts such as climate-related migration, intergenerational climate justice and pollution-related health risks. Conversely, Item 2 received the fewest suggestions, and these mainly concerned the inclusion of a definition of One Health and an additional response option (‘climate sceptical’). In Round 2, Item 11 generated the most proposed modifications, largely involving wording refinements and a clarification of the response options. Item 13 received only one suggestion about splitting a response category. Only Item 4 attracted more modification proposals than in Round 1. All the revisions accepted were incorporated into the subsequent questionnaire and re-evaluated during Round 3.

As the Delphi process progressed, qualitative feedback evolved from improving wording and conceptual clarity towards strengthening scientific precision, cultural relevance and contextual adaptation. One of the principal linguistic refinements was the replacement of the term ‘climate change’ with the term ‘climate disruption’ within the questionnaire items. This reflected experts’ preferences for terminology considered more conceptually precise in French. This modification was applied exclusively to the adapted CHANT items, and we have retained the term ‘climate change’ throughout this manuscript to ensure consistency with the original instrument and the international scientific literature. Our experts also expanded the questionnaire by incorporating concepts such as biodiversity loss, climate-related migration, gender inequalities and collective approaches to climate action. In the final round, recommendations focused on strengthening scientific rigour through clearer definitions, references to authoritative sources, greater consideration of structural determinants and an improved contextualisation of environmental sustainability within healthcare practice.

All the questionnaire items underwent one or more modifications from among the six predefined categories of linguistic and conceptual refinement. This resulted in progressive improvements in the translated instrument. In addition, one new item (Item 2) was added following Round 1’s expert consensus on the need to enhance the representation of the domain of *concern* in the final French-language version of the CHANT.

After the three Delphi rounds, the research team conducted a separate post-Delphi linguistic, semantic and cultural review. This review was not part of the Delphi process but constituted a final expert appraisal to ensure semantic equivalence, conceptual consistency and cultural appropriateness of the adapted French-language version. The study team agreed on the final wording of the 13-item French-language version following the final linguistic and semantic review (see Table 1).

As specified in the study protocol, exploratory analyses were conducted to examine potential associations between experts’ sociodemographic characteristics and their ratings. No significant sex-related differences were observed, whereas a limited number of significant associations were identified by educational level, profession and country of residence. Overall, these findings indicated comparable distributions of comprehension and relevance ratings between male and female participants, despite the predominance of females in the expert panel.

The Kruskal–Wallis test was used to compare ratings by experts’ educational level, profession and country of residence. In Round 1, statistically significant differences were found for the relevance of Item 6 by educational level (PhD, master’s, bachelor’s, specialisation or undergraduate) (H(2) = 9.65, *p* = 0.008, ε^2^ = 0.55) and for the comprehension of Item 6 by country (Switzerland, France, Belgium), (H(2) = 6.106, *p* = 0.047, ε^2^ = 0.29), with a large effect size. In Round 2, significant associations were found between the comprehension of items 11 and 12 and experts’ country, indicating large effect sizes (H(1) = 9.00, *p* = 0.003, ε^2^ = 0.62, for both items). Item 11′s relevance also varied by country (H(1) = 5.00, *p* = 0.025, ε^2^ = 0.31). Finally, in Round 3, the distribution of comprehension ratings for Item 5 also varied by country (H(3) = 9.031, *p* = 0.029, ε^2^ = 0.29), indicating a significant perceived difference. Item 11’s relevance varied according to experts’ professions (nurse, physician, allied health professional, health educator/clinical instructor or sustainability/environmental specialist) (H(7) = 16.00, *p* = 0.025, ε^2^ = 0.53). The significant associations observed between experts’ characteristics and item ratings suggest that professional experience, sociocultural contexts and personal backgrounds might all influence perceptions of climate-related concepts. Differences by profession may reflect varying levels of exposure to concepts involving sustainability and different professional priorities within healthcare practice. For example, experts with educational or environmental backgrounds may be more familiar with climate-related terminology than clinicians primarily focused on patient care activities. Similarly, differences associated with countries of residence may reflect contextual variations in healthcare systems, educational curricula, public policies and societal awareness of climate-related issues. Although Switzerland, France and Belgium share a common language, they differ in their approaches to sustainability and environmental health, which may influence interpretations of specific concepts.

Spearman’s rank correlations were calculated to examine relationships between items’ comprehension and relevance ratings, as well as between participants’ ages and questionnaire completion times. In Round 1, a significant positive correlation was identified between age and the comprehension of Item 8 (r_s_ = 0.55, *p* = 0.041), suggesting that older participants perceived this item as less comprehensible. No significant associations were observed between age and the comprehension of the other items. Likewise, no meaningful association was found for Item 11’s comprehension (r_s_ < 0.001, *p* = 1.000), indicating a negligible relationship. Regarding item relevance, a significant negative correlation was observed between Item 10 and questionnaire completion time (r_s_ = −0.73, *p* = 0.007), indicating that experts who rated this item as more relevant tended to complete the questionnaire more rapidly. In Round 2, a significant positive correlation emerged between the perceived relevance of Item 13 and completion time (r_s_ = 0.64, *p* = 0.047), suggesting that this item may have required greater reflection or cognitive processing. A significant negative association was also observed between age and the perceived relevance of Item 10 (r_s_ = −0.66, *p* = 0.037), indicating that older experts tended to assign it lower relevance ratings. In Round 3, a strong positive correlation was found between questionnaire completion time and comprehension of Item 12 (r_s_ = 0.75, *p* < 0.001), suggesting that participants requiring more time to complete the questionnaire may have considered this item in more detail.

Although several statistically significant associations were identified, these exploratory findings suggest only that perceptions of climate-related concepts differ across professional or sociodemographic characteristics. Given the limited sample sizes in each Delphi round and the number of statistical comparisons performed, these results should be interpreted with caution and regarded as hypothesis-generating. The final 13-item French-language version of the CHANT is provided in Supplementary File (the French version of the CHANT).

## 4. Discussion

**Figure 2:**
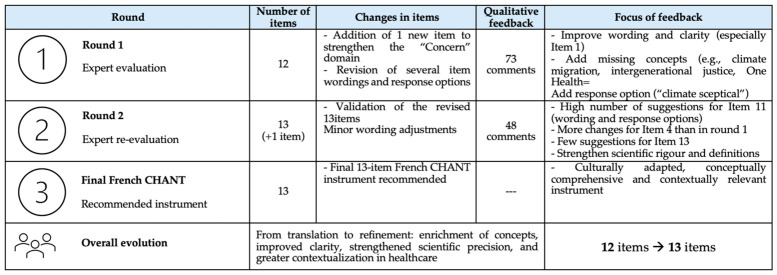
Evolution of the CHANT throughout our modified e-Delphi process.

Our modified e-Delphi survey facilitated the iterative refinement of a French-language adaptation of the CHANT. Analysis yielded consistently high content validity over the three rounds, with the overall S-CVI increasing and several items (e.g., 1, 5, 8–12) reaching perfect I-CVI scores by the final round. The final French-language version consists of 13 items, compared to the original 12-item English-language instrument. The addition of one expert-generated item strengthened the instrument’s representation of the domain of *concern*, thereby improving its content coverage while reflecting the principles of rigorous cross-cultural adaptation (Figure 2). Iterative feedback enhanced comprehension, most notably for Item 10, while items such as 6 and 1 exhibited fluctuations suggesting potential wording or conceptual issues. Item 11 consistently demonstrated high relevance, although this varied by country, underscoring cultural and contextual influences. As expected, qualitative suggestions decreased in successive rounds, reflecting growing conceptual clarity and converging expert opinions. Finally, one of the most important linguistic refinements concerned the replacement of ‘climate change’ with ‘climate disruption’ within the adapted questionnaire, reflecting experts’ preference for terminology perceived as more conceptually precise in French.

Sociodemographic analyses revealed differences by education, profession and country of residence, with Item 11 again emerging as context-sensitive. Correlations between age, response time and ratings suggested that complexity influenced engagement. These findings highlight the importance of tailoring tools to local sociocultural and healthcare contexts to maximise their validity. Our diverse, international, interprofessional panel—including nurses, physicians, allied health professionals, educators and sustainability experts from Switzerland, France and Belgium—strengthened the instrument’s content validity by ensuring multiple perspectives. Overall, this process improved the content validity and cultural adaptation of the French-language version of the CHANT, resulting in a content-valid interprofessional instrument for clinical practice and research in healthcare.

### 4.1. Implications for Practice

Assessing nurses’ eco-literacy is a critical step in designing evidence-based educational strategies that strengthen environmental competencies in healthcare. Embedding nursing practice within an eco-responsible framework requires integrating environmental considerations into clinical decision-making while safeguarding quality and safety. Educational interventions must therefore be efficient, tailored and contextually relevant so as to foster proactive, ethically grounded action. A systematic review by Portela Dos Santos et al. [28] identified three main types of interventions for enhancing evidence-based practice (EBP) competencies: multifaceted strategies involving mentoring, single-component approaches (often online) and structured programmes based on the five EBP steps. Similarly, recent studies have shown the effectiveness of web-based, video-based and virtual educational programmes in improving nurses’ climate awareness, activism and pro-environmental behaviours [29,30]. Instruments such as the CHANT can further support the integration of environmental imperatives into safe, patient-centred nursing practice.

### 4.2. Implications for Education

Systematically integrating sustainability, planetary health and eco-literacy into nursing education curricula is imperative at all levels—undergraduate, postgraduate and continuing professional development. The present study suggests that the CHANT could be a useful instrument for identifying educational needs related to climate change and sustainability among nurses and nursing students. Such information could support the design and evaluation of targeted educational interventions and inform future curriculum development.

### 4.3. Implications for Research

Variations in the comprehension and relevance of certain CHANT items, particularly Item 11, underscored the need to consider cultural and contextual factors when developing assessment tools. This aligns with the principles of implementation science, which recommend adapting instruments to local healthcare systems, educational models and environmental priorities to ensure validity and acceptability [31,32,33,34]. Iterative adaptation involving stakeholders is therefore essential to maintaining relevance, engagement and clarity, and ultimately supporting culturally sensitive, eco-centric health leadership. The Consolidated Framework for Implementation Research provides a structured approach to guide such processes through their pre-, peri-, and post-implementation phases [32,33,34]. Finally, the addition of a new item based on experts’ recommendations could influence the French-language CHANT’s psychometric properties and comparability with the original version. Although this modification aimed to improve content relevance and contextual adaptation, further psychometric validation is required. A subsequent study will therefore evaluate the factorial structure, construct validity, internal consistency and reliability of the adapted French-language CHANT.

### 4.4. Strengths and Limitations

Situating the CHANT within the Integrated Change Model provided a useful theoretical framework for interpreting the dimensions assessed during the content validation process. The modified e-Delphi approach generated 57 completed expert evaluations across three iterative rounds, enabling multidisciplinary input from healthcare and sustainability experts in Switzerland, France and Belgium. This process preserved participant anonymity while supporting the iterative refinement of the translated questionnaire and its comprehensibility, relevance and cultural appropriateness.

This study established the content validity of the French-language CHANT but did not constitute a full psychometric validation. Further research is required to evaluate its structural validity, construct validity, reliability, measurement invariance and other psychometric properties before broader implementation.

Although panel membership varied across rounds, this reflects the methodological characteristics of a modified e-Delphi designed for questionnaire translation, cultural adaptation and content validation, where maintaining comparable expertise is more important than retaining identical participants. Finally, the exploratory inferential analyses should be interpreted cautiously because of the small sample sizes and the absence of adjustments for multiple comparisons. These findings should therefore be considered hypothesis-generating and confirmed in future psychometric studies involving larger French-speaking samples.

## 5. Conclusions

The final French-language adaptation of the CHANT comprises 13 items, including one expert-generated item that strengthened the representation of the environmental concern domain. This study constitutes the first step in the cross-cultural adaptation of the CHANT by establishing its content validity through a rigorous modified e-Delphi process. The resulting French-language version demonstrated satisfactory content validity and cultural appropriateness for French-speaking healthcare professionals. However, additional psychometric studies are required to evaluate its structural validity, construct validity, reliability and other measurement properties before its routine use in research, education and clinical practice. Establishing a fully validated French-language CHANT will facilitate the assessment of eco-literacy, eco-responsibility, and eco-centrism, and support the development of evidence-based educational and implementation strategies to strengthen healthcare professionals’ competencies in planetary health.

## Figures and Tables

**Table 1:** The multi-step modified e-Delphi survey procedure.

**Round 1—Expert Evaluation**		
**Questions asked about each item.** **Original CHANT (12 items)**		
Comprehensibility assessment	Relevance assessment	Qualitative feedback
We invite you to critically evaluate each item before rating it. Is this item comprehensible?	We invite you to critically evaluate each item before rating it. Is this item relevant? (Likert scale from 1 to 4).	We also invite you to provide written comments to help us improve the relevance of the items in relation to the domain targeted. All feedback will be considered in subsequent rounds.
1 = comprehensible 2 = partially comprehensible 3 = not at all comprehensible	1 = irrelevant 2 = somewhat relevant 3 = relevant 4 = highly relevant	Experts’ suggestions were made in free text. Experts could propose additional topics/items.
**Round 2—Expert Re-Evaluation.** **Revised CHANT (13 items, including the newly added ‘concern’ item)**		
**Presentation of all the Round 1 items to ensure a comprehensive overview of the CHANT and to highlight the modifications made following Round 1′s expert feedback and the consensus reached.** **An additional item was included after Round 1 based on the experts’ suggestions.**		
Comprehensibility assessment	Relevance assessment	Qualitative feedback
Items and response options	We invite you to critically evaluate each item before rating it. Is this item relevant? (Likert scale from 1 to 4).	We also invite you to provide written comments to help us improve the relevance of the items in relation to the domain targeted. All feedback will be considered in subsequent rounds.
1 = comprehensible 2 = partially comprehensible 3 = not at all comprehensible	1 = irrelevant 2 = somewhat relevant 3 = relevant 4 = highly relevant	Experts’ suggestions were made in free text. Experts could propose additional topics/items.
**Round 3—Final Delphi Consensus.** **Final CHANT (13 items)**		
**The final questionnaire was resubmitted to the experts, although no further comments were possible.**		
Comprehensibility assessment	Relevance assessment	
Items and response options	We invite you to critically evaluate each item before rating it. Is this item relevant? (Likert scale from 1 to 4).	
1 = comprehensible 2 = partially comprehensible 3 = not at all comprehensible	1 = irrelevant 2 = somewhat relevant 3 = relevant 4 = highly relevant	
**Post-Delphi linguistic and semantic review**		
1. After integrating all the comments from the international panel of experts, the research team developed a final consensus on the item statements and response options. 2. Final linguistic review and consensus decision on the semantic and cultural equivalence of the adapted French-language version.		

**Table 2:** Sociodemographic and professional data on the panel of experts participating in the modified e-Delphi survey.

Sociodemographic Variables	Round 1 Experts (n = 17)	Round 2 Experts (n = 15)	Round 3 Experts (n = 25)
**Country of residence, n (%)**			
Switzerland	13 (76.5%)	13 (86.7%)	14 (56%)
France	2 (11.8%)	2 (13.3%)	10 (40%)
Belgium	2 (11.8%)	0 (0%)	1 (4%)
**Sex, n (%)**			
Male	7 (41.2%)	4 (26.7%)	7 (28%)
Female	10 (58.8%)	11 (73.3%)	18 (72%)
**Profession**			
Nurse	5 (29.4%)	5 (33.3%)	7 (28%)
Nursing student	0 (0%)	0 (0%)	1 (4%)
Midwife	0 (0%)	2 (13.3%)	0 (0%)
Physician	3 (17.6%)	3 (20.0%)	7 (28%)
Allied health professional	4 (23.5%)	0 (0)	1 (4%)
Health educator/clinical instructor	1 (5.9%)	1 (6.7%)	3 (12%)
Sustainability/environmental specialist	4 (23.5%)	3 (20.0%)	3 (12%)
Others	0 (0%)	1 (6.7%)	3 (12%)
**Level of education, n (%)**			
Bachelor’s degree	4 (23.5%)	9 (60.0%)	6 (24%)
Master’s degree	10 (58.8%)	3 (20.0%)	9 (36%)
PhD	2 (11.8%)	3 (20.0%)	6 (24%)
Continuing education (specialisation)	1 (5.9%)	0 (0%)	3 (12%)
Undergraduate	0 (0%)	0 (0%)	1 (4%)
**Age [years], med (IQR)**	40 (17.5)	43 (23)	42 (23)
**Age [years], min–max**	26–73	22–74	20–67
**Questionnaire completion time [minutes], med (IQR)**	21.9 (21.4)	25.47 (34.35)	10.6 (21.2)

**Table 3:** I-CVI and relevance across the three rounds.

	Round 1 I-CVI	Mean Relevance		Round 2 I-CVI	Mean Relevance	Round 3 I-CVI	Mean Relevance
**Item 1**	0.93	3.53	**Item 1**	0.91	3.11	1	3.5
**---**	**---**	**---**	**Item 2**	0.71	3.33	0.84	3
**Item 2**	0.93	3.13	**Item 3**	1	3.67	0.89	3.3
**Item 3**	0.79	3.07	**Item 4**	1	3.67	0.89	3.3
**Item 4**	0.93	3.43	**Item 5**	1	3.67	1	3.67
**Item 5**	1	3.79	**Item 6**	1	3.22	0.82	3.56
**Item 6**	1	3.31	**Item 7**	0.90	3.22	0.88	3.67
**Item 7**	0.92	3.64	**Item 8**	1	3.44	1	3.44
**Item 8**	1	3.38	**Item 9**	1	3.67	1	3.63
**Item 9**	1	3.36	**Item 10**	1	3.56	1	3.78
**Item 10**	0.86	3.14	**Item 11**	0.90	3.67	1	3.56
**Item 11**	0.87	3.36	**Item 12**	0.94	3.44	1	3.56
**Item 12**	0.86	3.07	**Item 13**	0.90	3.22	0.82	3.22

**Table 4:** Comprehension across the three rounds.

	Round 1				Round 2			Round 3		
	1	2	3		1	2	3	1	2	3
**Item 1**	100%	0%	0%	**Item 1**	100%	0%	0%	90%	10%	0%
**---**				**Item 2**	90%	10%	0%	100%	0%	0%
**Item 2**	78.6%	21.4%	0%	**Item 3**	100%	0%	0%	95%	5%	0%
**Item 3**	78.6%	21.4%	0%	**Item 4**	100%	0%	0%	68.4%	31.6%	0%
**Item 4**	71.4%	28.6%	0%	**Item 5**	90%	10%	0%	84.2%	15.8%	0%
**Item 5**	92.9%	0%	7.1%	**Item 6**	100%	0%	0%	81.3%	18.7%	0%
**Item 6**	92.9%	7.1%	0%	**Item 7**	80%	0%	20%	94.4%	5.6%	0%
**Item 7**	85.7%	7.1%	7.1%	**Item 8**	100%	0%	0%	82.4%	17.6%	0%
**Item 8**	85.7%	7.1%	7.1%	**Item 9**	100%	0%	0%	93.8%	6.2%	0%
**Item 9**	71.4%	21.4%	7.1%	**Item 10**	100%	0%	0%	100%	0%	0%
**Item 10**	78.6%	21.4%	0%	**Item 11**	90%	10%	0%	94.8%	5.2%	0%
**Item 11**	85.7%	14.3%	0%	**Item 12**	90%	10%	0%	94.1%	5.9%	0%
**Item 12**	92.9%	7.1%	0%	**Item 13**	100%	0%	0%	94.4%	5.6%	0%

For comprehension, the number of ratings at 1, 2 or 3 is shown as a percentage.

**Table 5:** Experts’ comments and suggestions.

Item	Round	Re (n)	Add (n)	Experts’ Suggestions
1	1	2	7	Add concepts and definitions
	2	2	4	Clarify gender-related impacts; add a response option; add scientific references
2	1	0	3	Add a definition of One Health; add response options
	2	2	0	Make minor wording refinements
3	1	6	3	Clarify climate/weather distinction; add concepts of ‘landslide’ and ‘heatwave’
	2	2	1	Add a new concept
4	1	2	3	Define the refinement; clarify the concept; provide illustrative example
	2	3	0	Further simplify wording
5	1	4	3	Add and revise concepts
	2	3	3	Add concepts and response options
6	1	4	1	Modify item wording
	2	3	0	Simplify the item and response wording
7	1	4	2	Add response options
	2	3	0	Make minor wording modifications
8	1	5	0	Make minor wording refinements
	2	3	2	Add response options
9	1	6	0	Make wording refinements
	2	1	2	Add concepts
10	1	6	2	Add concepts
	2	2	0	Make minor wording refinements
11	1	4	2	Add response options
	2	6	2	Clarify response categories
12	1	2	2	Add concepts
	2	2	1	Add concept
13	2	1	0	Split a response category
Total		78	43	

Re = reformulation; Add = addition of a term, definition, concept or other.

## Data Availability

The original contributions presented in this study are included in the article/Supplementary Material. Further inquiries can be directed to the corresponding author.

## Supplementary Materials

The following supporting information can be downloaded at: https://www.mdpi.com/article/10.3390/nursrep16070244/s1. The French version of the CHANT.

## Public Involvement Statement

There was no public involvement in any aspect of this research.

## Guidelines and Standards Statement

This manuscript was drafted in accordance with the Conducting and Reporting Delphi Studies (CREDES) guidelines for observational research.
