# Supplementary material for: Content Validity of the CHANT’s French-Language Translation and Cultural Adaptation: A Modified E-Delphi Study
Source: Nurs Rep. 2026 Jul 15;16(7):244. doi: 10.3390/nursrep16070244 (PMC13415604; doi:10.3390/nursrep16070244)
Supplement: Supplementary file 1 [file nursrep-16-00244-s001.zip › nursrep-4376698-supplementary.pdf]

Item	Échelle
1. Sensibilisation 1	
Veuillez indiquer à quel point vous êtes au courant des affirmations suivantes, fondées sur des preuves ?<br>o La planète s'est réchauffée de manière significative depuis les années 1850, provoquant un dérèglement climatique<br>o Ce dérèglement est en grande partie attribuable aux activités humaines développées depuis la révolution industrielle<br>o La combustion d'énergies fossiles (charbon, pétrole, gaz), la déforestation et l'agriculture intensive libèrent d'importantes quantités de gaz à effet de serre, renforçant ainsi l'effet de serre naturel.<br>o En 2021, les émissions de gaz à effet de serre du secteur de la santé ont augmenté de 9,5 % par rapport à 2020, représentant 4,6% des émissions mondiales (The Lancet, 2024)<br>o Le dérèglement climatique accroît la probabilité de problèmes de santé tels que les coups de chaleur, l'exacerbation de l'asthme, maladie de Lyme, les maladies cardiovasculaires, les problématiques de santé mentales, etc.<br>o Les populations vulnérables, telles que les personnes très jeunes ou très âgées, et d'autres groupes à risque (personnes sans domicile fixe ou vivant dans la pauvreté, personnes atteintes de maladies chroniques, femmes, etc.) subissent davantage les effets délétères du dérèglement climatique sur la santé<br>o L'effondrement de la biodiversité est de plus en plus intense et rapide. Ill est lié aux activités humaines industrielles et d'urbanisation. La première cause est la destruction des habitats, puis l'exploitation et le dérèglement climatique<br>o La crise climatique augmente les migrations forcées en rendant certaines régions inhabitables (cyclones, inondations, incendie, etc.). La migration concerne surtout les populations les moins responsables du dérèglement climatique (Senn et al, 2022)<br>o Le dérèglement climatique frappe plus durement les femmes, surtout dans les pays pauvres. La raréfaction des ressources, les déplacements forcés et la dégradation de l'environnement, les exposent davantage à la pauvreté, aux la violences sexistes (comme les mariages forcés ou les violences sexuelles) et voient leur accès à l'éducation, à la santé et aux ressources limité (OCDE, 2021).	Échelle de sensibilisation :<br><br>1 = Pas du tout familier<br>2 = Peu familier<br>3 = Un peu familier<br>4 = Moyennement familier<br>5 = Extrêmement familier
2. Sensibilisation 2	
J'ai entendu parler du dérèglement climatique par les sources suivantes (cochez toutes les cases correspondantes) :<br>o Presse écrite<br>o Journal télévisé<br>o Médias sociaux<br>o Internet<br>o Cours professionnels<br>o Organisations professionnelles<br>o Amis et/ou famille<br>o Vie associative et/ou de quartier<br>o Autre : ________________________<br>Je n'ai jamais entendu parler du dérèglement climatique	
3. Expérience 1	
À quelle fréquence avez-vous remarqué les phénomènes physiques et événements météorologiques suivants (ou leurs conséquences) dans votre région ?<br>o Températures extrêmes<br>o Fortes précipitations<br>o Sécheresse<br>o Inondations<br>o Ouragans et tempêtes<br>o Incendies de forêt<br>o Glissement de terrain<br>o Fonte des glaciers<br>o Monté du niveau de la mer<br>o Éboulements	Échelle d'expérience :<br><br>1 = Jamais<br>2 = Rarement (1x/10 ans)<br>3= Occasionnellement (1x/5 ans)<br>4 = Fréquemment (1x/an)<br>5 = Très souvent (> 1x/an)

4. Expérience 2	
Les autorités sanitaires identifient plusieurs problèmes de santé aggravés par le dérèglement climatique. Pour chaque item, à quelle fréquence observez-vous ces affections ?<br><br>**2a : Mes patient.es /client.es**<br>o Maladies respiratoires : asthme, allergies, BPCO, etc.<br>o Maladies transmises par des vecteurs : maladie de Lyme, encéphalite à tique, malaria, etc.<br>o Symptômes dus ou exacerbés par le stress thermique (îlots de chaleur, canicule, vagues de froid, etc.) : coup de chaleur, déshydratation, épuisement, hypothermie, engelure, etc.<br>o Traumatismes physiques liés à des événements climatiques extrêmes : tempêtes, inondations, incendies, éboulements, etc.<br>o Troubles de santé mentale : dépression, anxiété, stress post-traumatique, angoisse face à l'avenir, sentiment d'impuissance ou de colère<br><br>**2b : Moi-même / mon entourage proche**<br>o Maladies respiratoires : asthme, allergies, BPCO, etc.<br>o Maladies transmises par des vecteurs : maladie de Lyme, encéphalite à tique, malaria, etc.<br>o Symptômes dus ou exacerbés par le stress thermique (îlots de chaleur, canicule, vagues de froid, etc.) : coup de chaleur, déshydratation, épuisement, hypothermie, engelure, etc.<br>o Traumatismes physiques liés à des événements climatiques extrêmes : tempêtes, inondations, incendies, éboulements, etc.<br>o Troubles de santé mentale : dépression, anxiété, stress post-traumatique, angoisse face à l'avenir, sentiment d'impuissance ou de colère<br><br>**2c : Cas que j'ai lus ou personnes dont j'ai entendu parler, mais que je ne connais pas personnellement**<br>o Maladies respiratoires : asthme, allergies, BPCO, etc.<br>o Maladies transmises par des vecteurs : maladie de Lyme, encéphalite à tique, malaria, etc.<br>o Symptômes dus ou exacerbés par le stress thermique (îlots de chaleur, canicule, vagues de froid, etc.) : coup de chaleur, déshydratation, épuisement, hypothermie, engelure, etc.<br>o Traumatismes physiques liés à des événements climatiques extrêmes : tempêtes, inondations, incendies, éboulements, etc.	Échelle d'expérience :<br><br>1 = Jamais<br>2 = Rarement (1x/10 ans)<br>3= Occasionnellement (1x/ 5ans)<br>4 = Fréquemment (1x/an)<br>5 = Très souvent (> 1x/an)
5. Préoccupation 1	
Dans quelles mesures les conséquences du dérèglement climatique, qu'elles soient directes ou indirectes, vous préoccupent-elles ?<br>o Impacts sur votre santé et celle de vos proches<br>o Impacts sur la santé de la population en général<br>o Impact sur mon travail / ma charge de travail<br>o Difficultés d'accès aux soins pour les patients<br>o Conséquences économiques et sociales personnelles (perte d'emploi, précarité, augmentation du coût de la vie, déplacements forcés, etc.)<br>o Conséquences économiques et sociales collectives (sécheresses, inondations, déstabilisation de régions entières, migration de populations, tensions sur les infrastructures, etc.)<br>o Conséquences financières (reconstruction après des catastrophes naturelles, augmentation des coûts de santé, réparations, etc.)<br>o Risques liés aux pénuries alimentaires ou d'eau (manque de ressources, baisse de qualité ou de quantité, dépendance accrue aux importations, etc.)<br>o Risques socio-politiques (conflits liés à l'accès aux ressources, migrations forcées, tensions géopolitiques, etc.)	Échelle de préoccupation :<br><br>1 = Pas du tout<br>2 = Légèrement<br>3 = Un peu<br>4 = Modérément<br>5 = Extrêmement

- o Conséquences pour les générations futures (dégradation des conditions de vie, raréfaction des ressources, augmentation des inégalités, etc.)
- o Impacts sur les écosystèmes et la biodiversité (faune, flore, forêts, océans, sols, etc.)
- o Autres : ________________________

6. Préoccupation 2

Quelles émotions ressentez-vous lorsque le sujet du dérèglement climatique apparaît dans une discussion ou dans les médias ? (Écrivez les émotions qui vous viennent en tête)

Émotions : ____________________________________

7. Optimisme 1

Dans quelle mesure êtes-vous optimiste quant à la capacité de l'humain à :<br>o S'adapter efficacement aux impacts du dérèglement climatique ?<br>o Atténuer l'aggravation du dérèglement climatique en transformant ses modes de vie individuels ?<br>o Atténuer l'aggravation du dérèglement climatique par des modifications systémiques et politiques ?	Échelle d'optimisme :<br><br>1 = Pas du tout<br>2 = Légèrement<br>3 = Un peu<br>4 = Moyennement<br>5 = Extrêmement

8. Motivation 1

D'après le *Lancet Countdown*, le secteur de la santé est responsable d'environ 4,6 % des émissions mondiales de gaz à effet de serre. À titre de comparaison, cela équivaut à peu près aux émissions totales de la Russie en 2023, qui représentaient environ 5 % des émissions mondiales (EDGAR Community GHG Database / Commission européenne, JRC et Agence internationale de l'énergie, 2024). Pour chaque catégorie, veuillez indiquer dans quelle mesure les affirmations suivantes sont vraies pour vous :<br><br>**Adapter ma pratique pour réduire l'impact environnemental des soins :**<br>o J'agis déjà en ce sens<br>o Je souhaite agir mais ne sais pas par où commencer<br>o Je souhaite agir mais crains de nuire à la qualité des soins<br>o Je n'ai pas les ressources nécessaires (temps, connaissances, obstacles à l'action, etc.)<br>o Je ne ressens pas le besoin de changer ma pratique<br><br>**Sensibiliser mon entourage professionnel aux liens santé- environnement**<br>o Je souhaite informer et mobiliser mon entourage professionnel et mes patient.e.s<br>o Je souhaite participer à des actions collectives<br>o Je n'ai pas les ressources pour le faire (temps, connaissances, obstacles à l'action, etc.).<br>o Ce n'est pas une priorité pour moi<br><br>**Me préparer aux impacts santé du changement climatique**<br>o Je souhaite adapter ma pratique aux nouvelles pathologies liées au climat<br>o Je souhaite renforcer la prévention pour limiter les nouvelles pathologies liées au climat<br>o Je souhaite aider à rendre le système de soins plus résilient<br>o Je n'ai pas les ressources pour me préparer (temps, connaissances, obstacles à l'action, etc.)<br>o Je ne vois pas cela comme un enjeu prioritaire	Échelle de motivation<br>1 = Pas du tout vrai pour moi<br>2 = Quelque peu vrai pour moi<br>3 = Neutre<br>4 = Vrai pour moi<br>5 = Très vrai pour moi<br>Non applicable

9. Motivation 2

Quelles sont les raisons qui vous poussent à agir pour limiter le dérèglement climatique ? (Pour chaque catégorie, cochez toutes les cases qui s'appliquent)

Nature et environnement

- o Mon expérience personnelle avec la nature
- o Dégradation de la qualité de l'air et de l'eau
- o Perte de biodiversité
- o Catastrophes climatiques
- o Montée des eaux qui menacent les zones côtières

Santé humaine et des écosystème (One Health)

- Impact sur ma santé / mes proches
- Nouvelles maladies
- Anxiété et incertitude liée au climat
- Mon rôle de soignant.e : protéger la santé globale, humaine et environnementale

Économie et société

- Coûts des crises climatiques
- Risques pour les emplois / secteurs clés (agriculture, tourisme, pêches, etc.)
- Territoires menacés
- Inégalités sociales et injustice climatique

Envie d'un modèle post-capitaliste/post-croissance (richesses et pouvoir partagés, fin de la surconsommation, etc.)

10 Motivation 3

Quelles sont les raisons qui limitent votre engagement dans la lutte contre le dérèglement climatique ?
(Pour chaque catégorie, cochez toutes les cases qui s'appliquent)

Contraintes personnelles

- Je manque de temps
- J'ai d'autres priorités
- Je consacre mon énergie à d'autres causes

Ressources et capacités

- Je pense que les actions pour le climat sont trop coûteuses
- Je ne sais pas par où commencer
- Le sujet me semble trop complexe
- Je manque de connaissances ou de moyens d'agir efficacement

Opinions et perceptions

- Je doute que l'action individuelle puisse vraiment changer les choses
- Je pense que c'est d'abord aux gouvernements et aux entreprises d'agir

Freins externes

- Je crains que la transition nuise à l'économie ou à l'emploi
- Je crains que cela provoque des tensions ou des conflits dans mon pays
- Je ne soutiens pas certaines mesures proposées politiquement

Niveau d'engagement

- J'agis déjà selon mes possibilités
- Je ne souhaite pas m'impliquer davantage
- Autre : ___________________________________

11. Comportement 1

À quelle fréquence adoptez-vous des pratiques écoresponsables <u>dans votre quotidien</u> ?<br><br>(L'action pour le climat est étroitement liée à d'autre enjeux comme la biodiversité, la justice sociale, l'emploi ou l'éducation. Chacun n'a pas les mêmes ressources ou possibilités pour agir mais de nombreuses formes d'action existent. Ensemble, elles peuvent contribuer à un changement plus global et collectif.)<br><br>o Réduire sa consommation d'énergie (chauffage, éclairage, appareils électriques)<br>o Utiliser des équipements économes en énergie et privilégier les énergies renouvelables<br>o Limiter l'usage de la voiture et privilégier les alternatives (vélo, marche, transports en commun)<br>o Réduire les trajets en avion lorsque c'est possible<br>o Privilégier une alimentation durable (locale, de saison, moins transformée, moins de viande)	Échelle de comportement (1 et 2) :<br><br>1 = Jamais<br>2 = Rarement (1x/an)<br>3 = Occasionnellement (1x/mois)<br>4 = Fréquemment (1x/semaine)<br>5 = Quotidiennement

o Limiter les achats et favoriser le recyclage<br>o Limiter le gaspillage<br>o Soutenir des placements bancaires éco-responsables<br>o Voter et soutenir des politiques environnementales	
12. Comportement 2	
À quelle fréquence adoptez-vous des pratiques écoresponsables dans votre environnement de travail ?<br>o Limiter la consommation d'énergie au travail (réduction du chauffage/climatisation, extinction des équipements inutilisés, utilisation des escaliers plutôt que l'ascenseur)<br>o Proposer ou soutenir des mesures pour améliorer l'efficacité énergétique sur son lieu de travail<br>o Privilégier les modes de transport durables pour se rendre au travail (marche, vélo, transports en commun, covoiturage)<br>o Limiter les déplacements professionnels à fort impact carbone (réduction des trajets en avion, recours aux visioconférences, etc.)<br>o Limiter le gaspillage de matériel et de ressources (papier, plastique, linge, matériel de soin)<br>o Encourager son employeur à adopter des politiques réduisant l'empreinte carbone du secteur<br>o Adopter des pratiques de consommation et de soins responsables sur son lieu de travail (réduction des emballages, alternatives au plastique à usage unique, prescription raisonnée selon les principes « Moins, Autrement, Mieux », médecine intégrative, dispensation adaptée des médicaments, etc.)	Échelle de comportement (1 et 2) :<br><br>1 = Jamais<br>2 = Rarement (1x/an)<br>3 = Occasionnellement (1x/mois)<br>4 = Fréquemment (1x/semaine)<br>5 = Quotidiennement
13. Comportement 3	
À quelle fréquence échangez-vous (en personne, par téléphone, par courriel, par lettre, etc.) sur le dérèglement climatique et la santé avec ces groupes ou individus ?<br>o Collègues<br>o Patient·es / client·es<br>o Ami.e.s /Voisin·es<br>o Famille<br>o Responsables politiques<br>o Associations locales<br>o Autres : ___________________________	Échelle de comportement (3) :<br>1 = Jamais<br>2 = Tous les ans<br>3 = 2 à 3 fois par an<br>4 = Tous les mois<br>5 = Toutes les semaines
